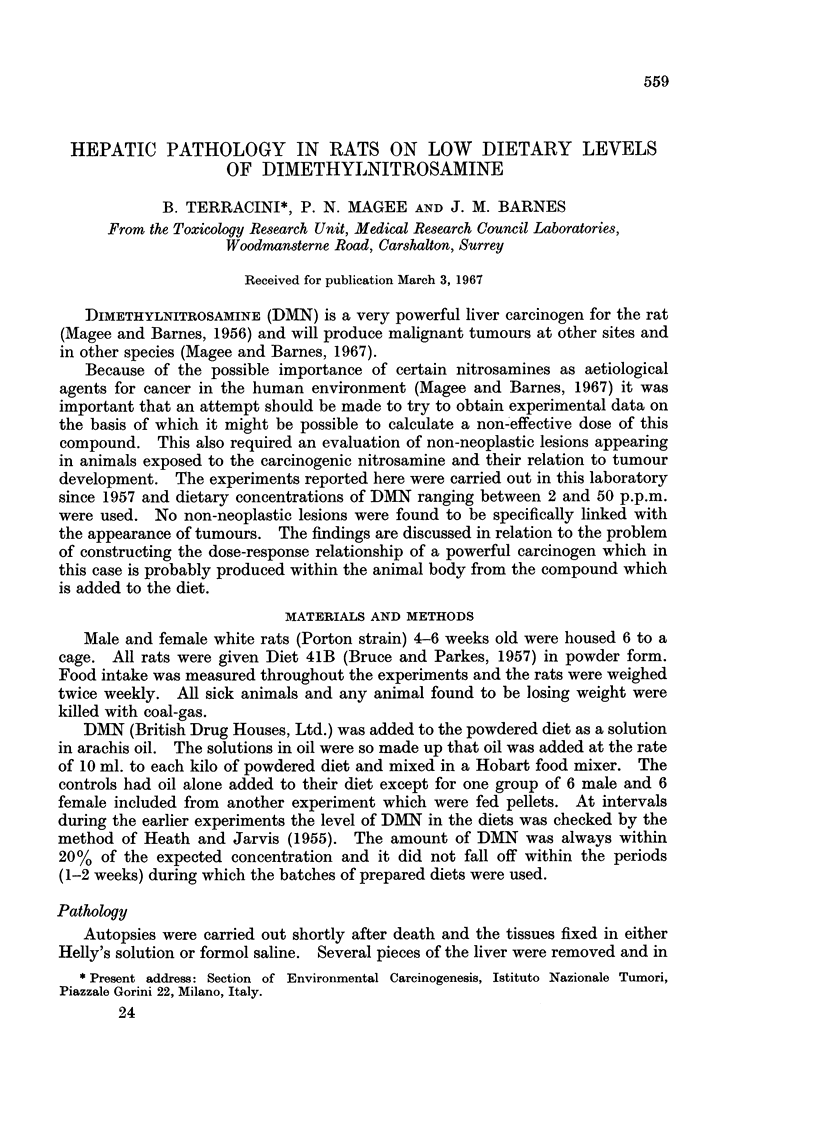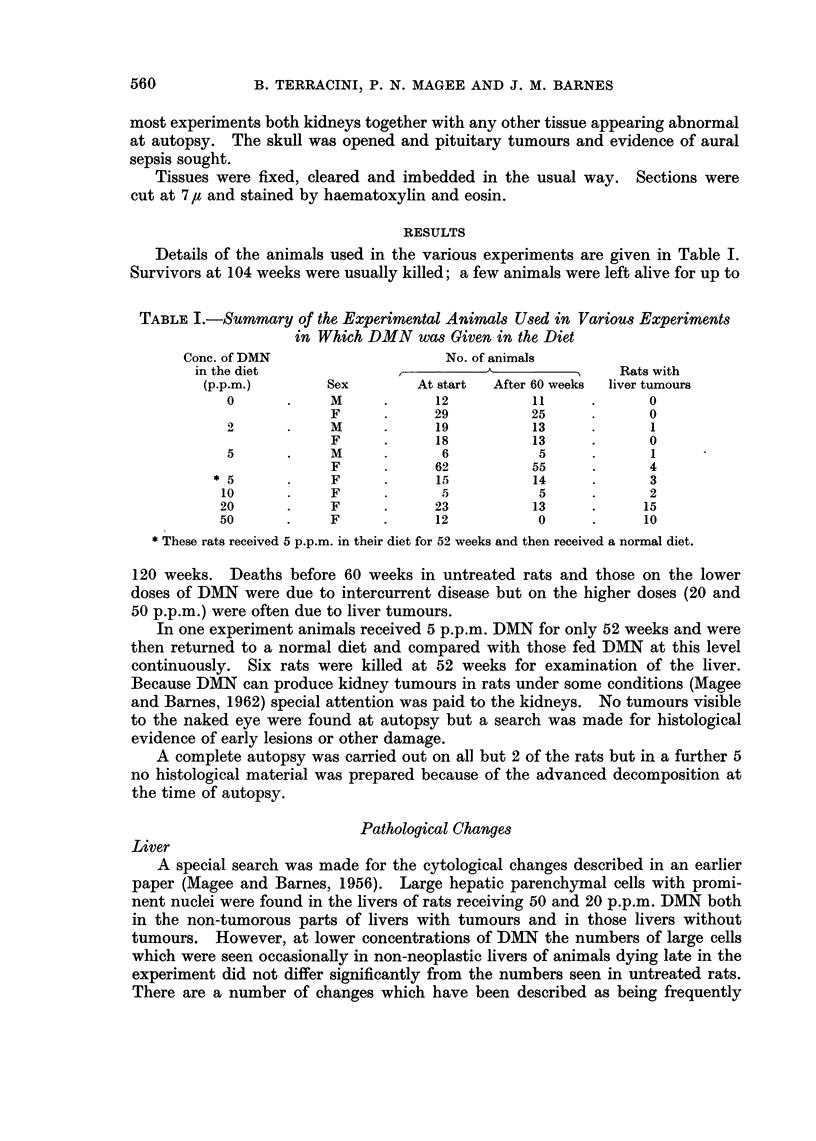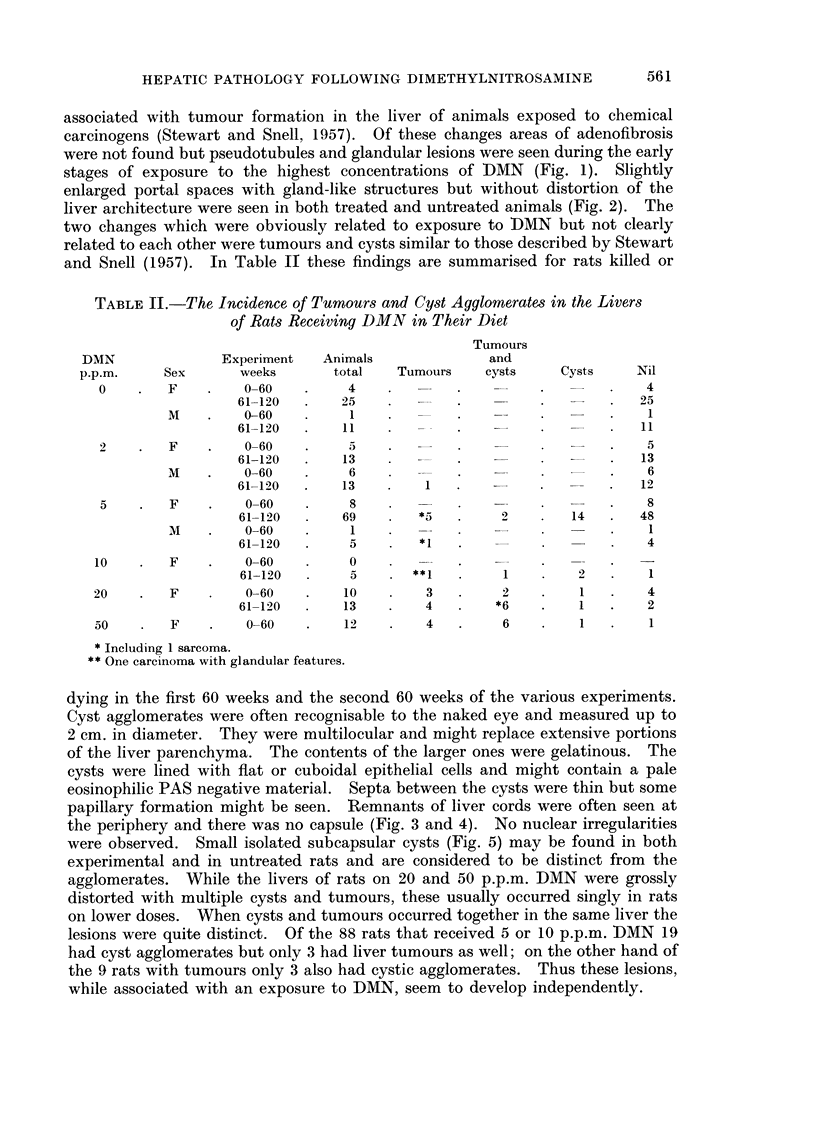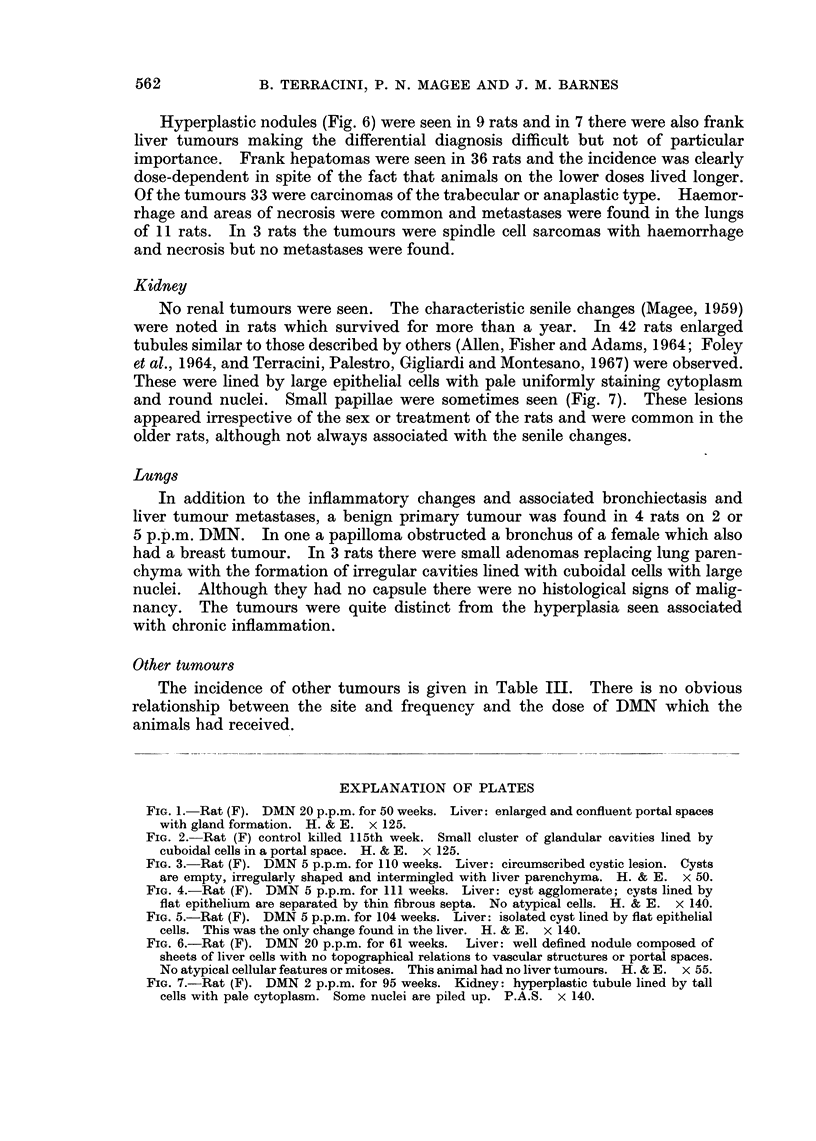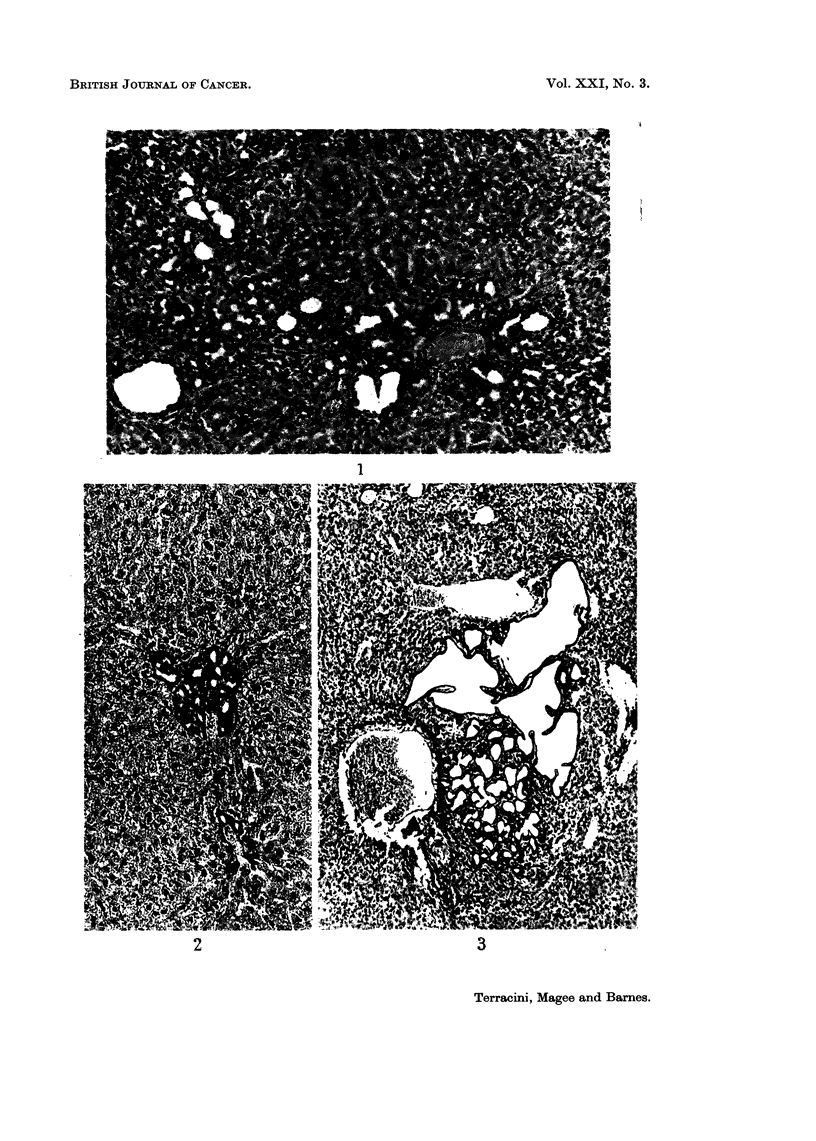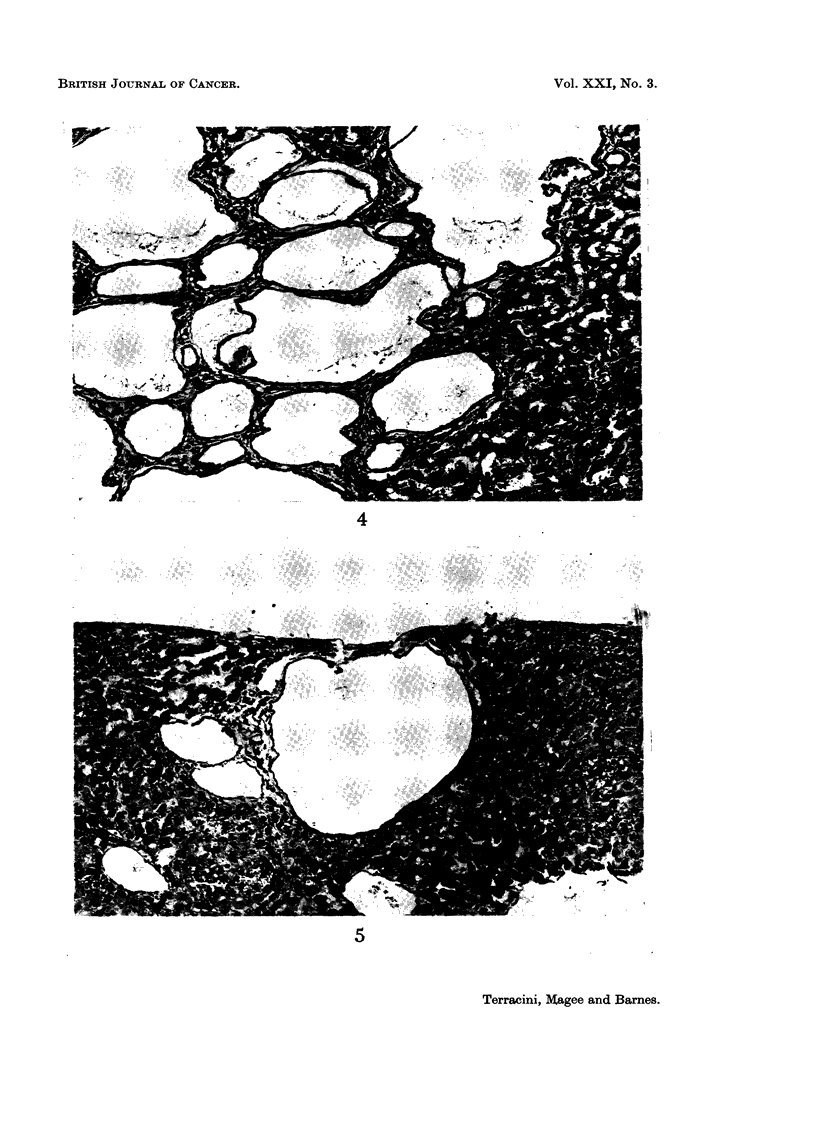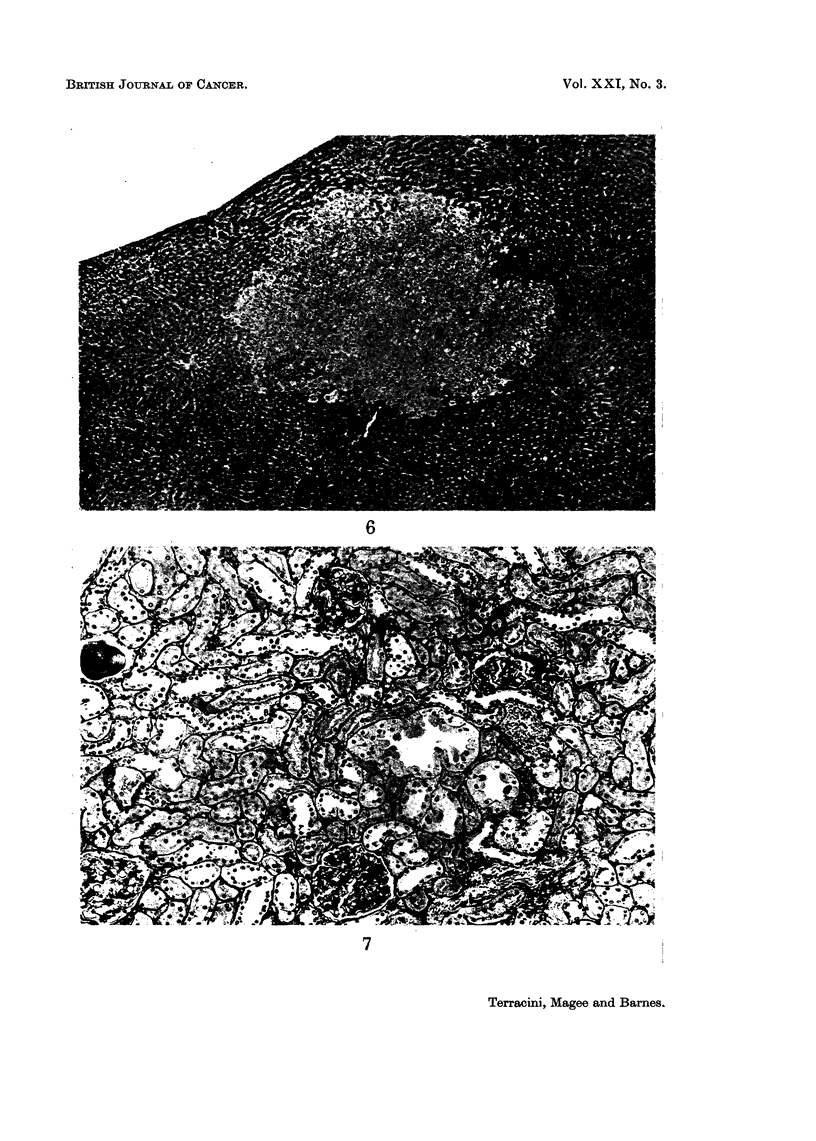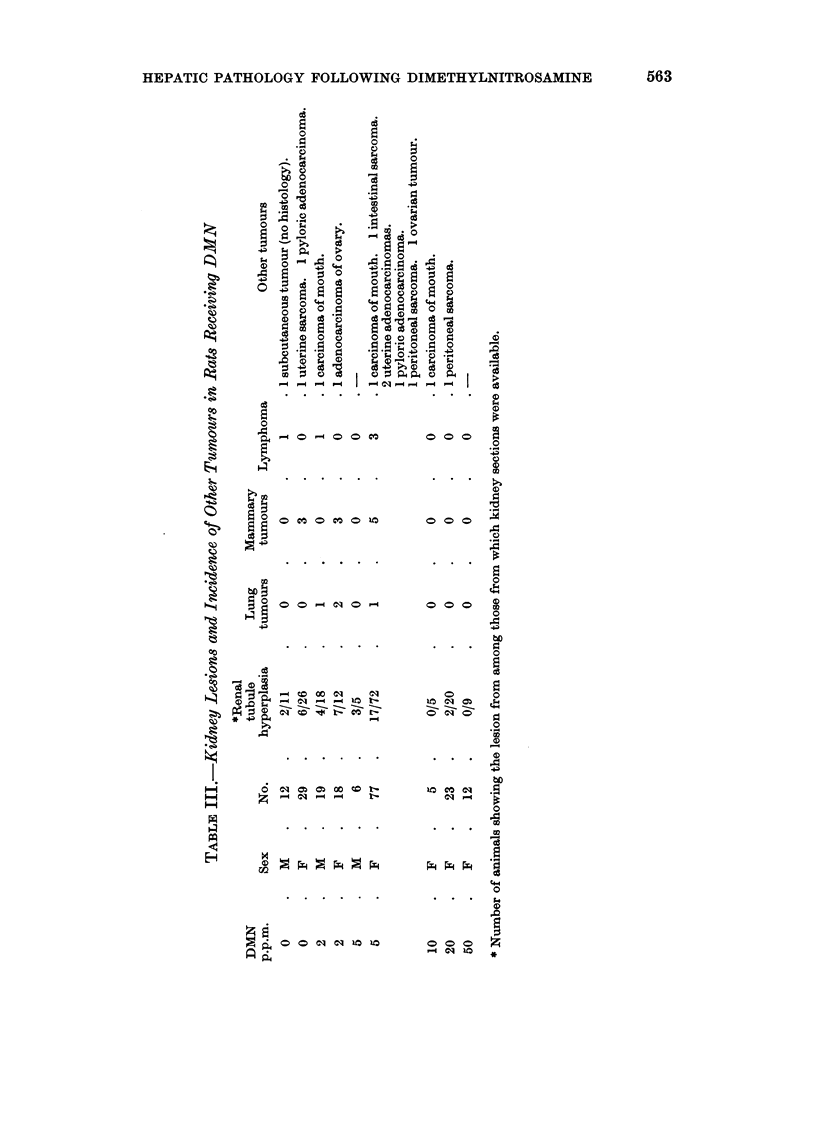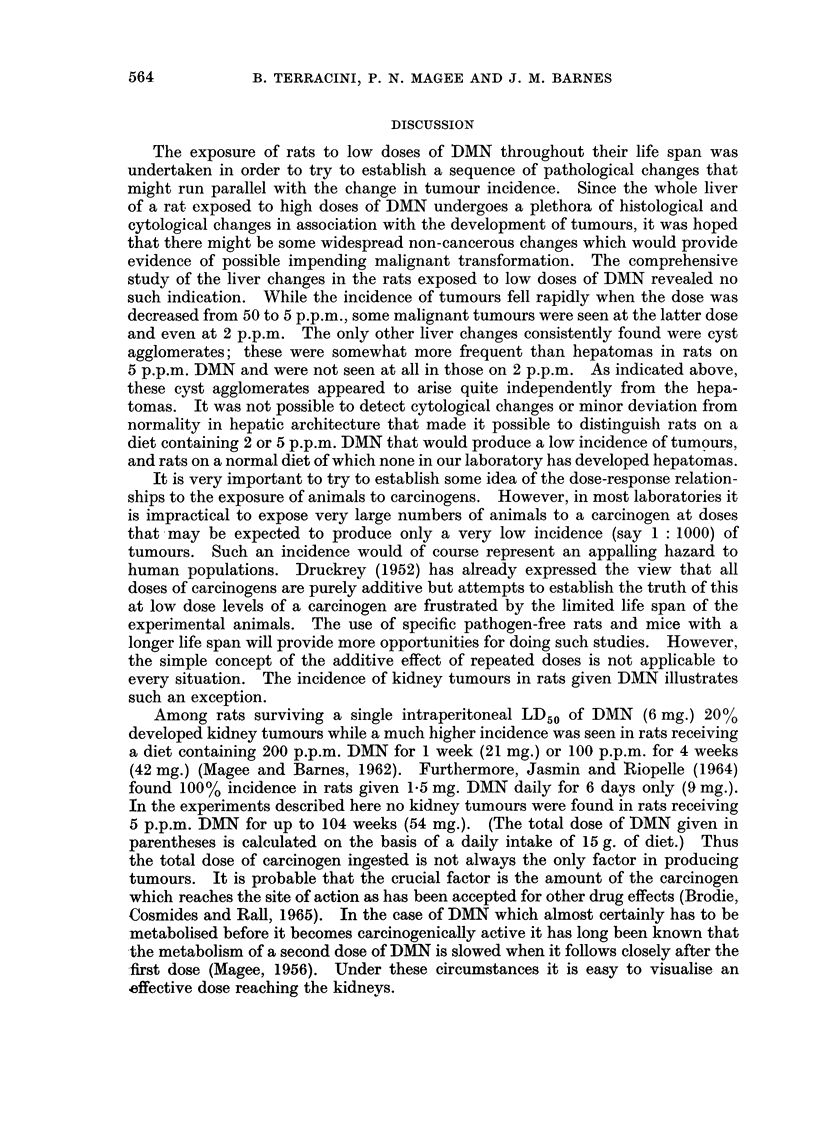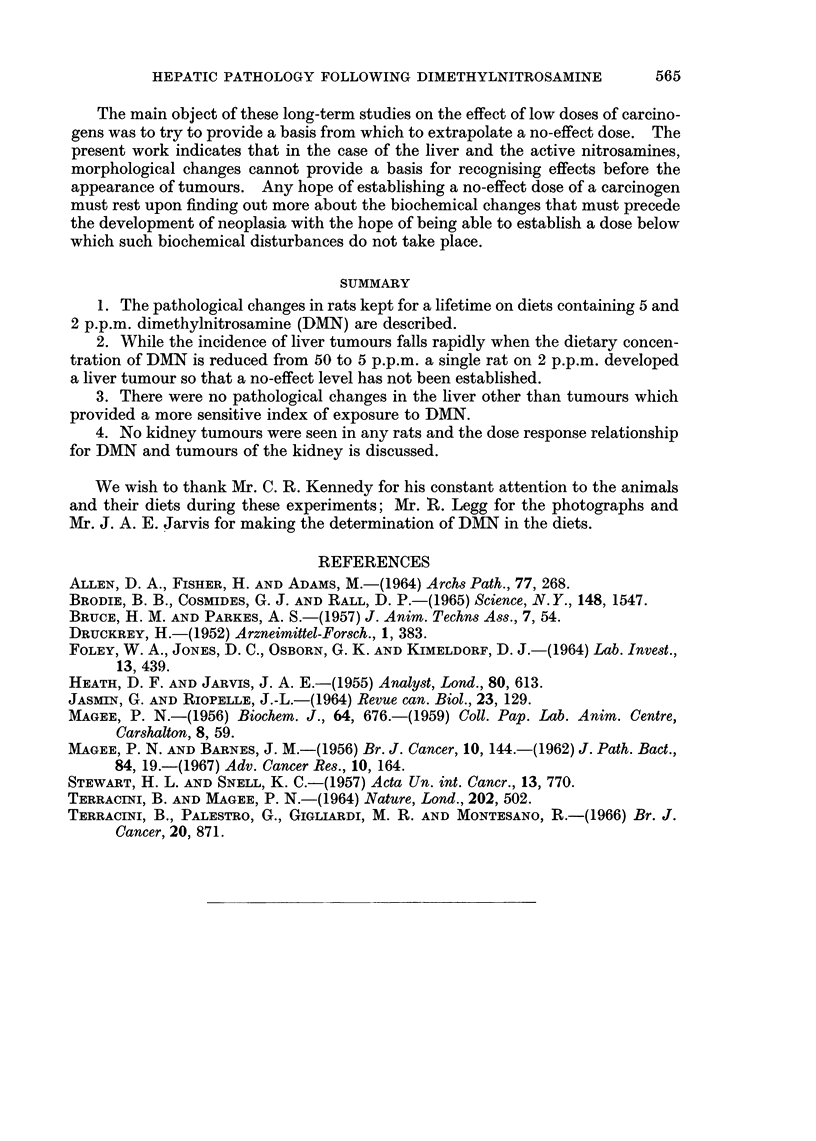# Hepatic pathology in rats on low dietary levels of dimethylnitrosamine.

**DOI:** 10.1038/bjc.1967.65

**Published:** 1967-09

**Authors:** B. Terracini, P. N. Magee, J. M. Barnes

## Abstract

**Images:**


### HEPATIC PATHOLOGY IN RATS ON LOW DIETARY LEVELS OF DIMETHYLNITROSAMINE

B. TERRACINI*, P. N. MAGEE AND J. M. BARNES
*From the Toxicology Research Unit, Medical Research Council Laboratories, Woodmansterne Road, Carshalton, Surrey*

Received for publication March 3, 1967

DIMETHYLNITROSAMINE (DMN) is a very powerful liver carcinogen for the rat (Magee and Barnes, 1956) and will produce malignant tumours at other sites and in other species (Magee and Barnes, 1967).

Because of the possible importance of certain nitrosamines as aetiological agents for cancer in the human environment (Magee and Barnes, 1967) it was important that an attempt should be made to try to obtain experimental data on the basis of which it might be possible to calculate a non-effective dose of this compound. This also required an evaluation of non-neoplastic lesions appearing in animals exposed to the carcinogenic nitrosamine and their relation to tumour development. The experiments reported here were carried out in this laboratory since 1957 and dietary concentrations of DMN ranging between 2 and 50 p.p.m. were used. No non-neoplastic lesions were found to be specifically linked with the appearance of tumours. The findings are discussed in relation to the problem of constructing the dose-response relationship of a powerful carcinogen which in this case is probably produced within the animal body from the compound which is added to the diet.

### MATERIALS AND METHODS

Male and female white rats (Porton strain) 4–6 weeks old were housed 6 to a cage. All rats were given Diet 41B (Bruce and Parkes, 1957) in powder form. Food intake was measured throughout the experiments and the rats were weighed twice weekly. All sick animals and any animal found to be losing weight were killed with coal-gas.

DMN (British Drug Houses, Ltd.) was added to the powdered diet as a solution in arachis oil. The solutions in oil were so made up that oil was added at the rate of 10 ml. to each kilo of powdered diet and mixed in a Hobart food mixer. The controls had oil alone added to their diet except for one group of 6 male and 6 female included from another experiment which were fed pellets. At intervals during the earlier experiments the level of DMN in the diets was checked by the method of Heath and Jarvis (1955). The amount of DMN was always within 20% of the expected concentration and it did not fall off within the periods (1–2 weeks) during which the batches of prepared diets were used.

### *Pathology*

Autopsies were carried out shortly after death and the tissues fixed in either Helly's solution or formol saline. Several pieces of the liver were removed and in

* Present address: Section of Environmental Carcinogenesis, Istituto Nazionale Tumori, Piazzale Gorini 22, Milano, Italy.

most experiments both kidneys together with any other tissue appearing abnormal at autopsy. The skull was opened and pituitary tumours and evidence of aural sepsis sought.

Tissues were fixed, cleared and imbedded in the usual way. Sections were cut at 7 $\mu$ and stained by haematoxylin and eosin.

### RESULTS

Details of the animals used in the various experiments are given in Table I. Survivors at 104 weeks were usually killed; a few animals were left alive for up to 120 weeks. Deaths before 60 weeks in untreated rats and those on the lower doses of DMN were due to intercurrent disease but on the higher doses (20 and 50 p.p.m.) were often due to liver tumours.

TABLE I.—*Summary of the Experimental Animals Used in Various Experiments in Which DMN was Given in the Diet*

| Conc. of DMN in the diet (p.p.m.) | Sex | No. of animals At start | No. of animals After 60 weeks | Rats with liver tumours |
|---|---|---|---|---|
| 0 | M | 12 | 11 | 0 |
| | F | 29 | 25 | 0 |
| 2 | M | 19 | 13 | 1 |
| | F | 18 | 13 | 0 |
| 5 | M | 6 | 5 | 1 |
| | F | 62 | 55 | 4 |
| * 5 | F | 15 | 14 | 3 |
| 10 | F | 5 | 5 | 2 |
| 20 | F | 23 | 13 | 15 |
| 50 | F | 12 | 0 | 10 |

* These rats received 5 p.p.m. in their diet for 52 weeks and then received a normal diet.

In one experiment animals received 5 p.p.m. DMN for only 52 weeks and were then returned to a normal diet and compared with those fed DMN at this level continuously. Six rats were killed at 52 weeks for examination of the liver. Because DMN can produce kidney tumours in rats under some conditions (Magee and Barnes, 1962) special attention was paid to the kidneys. No tumours visible to the naked eye were found at autopsy but a search was made for histological evidence of early lesions or other damage.

A complete autopsy was carried out on all but 2 of the rats but in a further 5 no histological material was prepared because of the advanced decomposition at the time of autopsy.

### *Pathological Changes*

*Liver*

A special search was made for the cytological changes described in an earlier paper (Magee and Barnes, 1956). Large hepatic parenchymal cells with prominent nuclei were found in the livers of rats receiving 50 and 20 p.p.m. DMN both in the non-tumorous parts of livers with tumours and in those livers without tumours. However, at lower concentrations of DMN the numbers of large cells which were seen occasionally in non-neoplastic livers of animals dying late in the experiment did not differ significantly from the numbers seen in untreated rats. There are a number of changes which have been described as being frequently associated with tumour formation in the liver of animals exposed to chemical carcinogens (Stewart and Snell, 1957). Of these changes areas of adenofibrosis were not found but pseudotubules and glandular lesions were seen during the early stages of exposure to the highest concentrations of DMN (Fig. 1). Slightly enlarged portal spaces with gland-like structures but without distortion of the liver architecture were seen in both treated and untreated animals (Fig. 2). The two changes which were obviously related to exposure to DMN but not clearly related to each other were tumours and cysts similar to those described by Stewart and Snell (1957). In Table II these findings are summarised for rats killed or

TABLE II.—*The Incidence of Tumours and Cyst Agglomerates in the Livers of Rats Receiving DMN in Their Diet*

| DMN p.p.m. | Sex | Experiment weeks | Animals total | Tumours | Tumours and cysts | Cysts | Nil |
|---|---|---|---|---|---|---|---|
| 0 | F | 0–60 | 4 | — | — | — | 4 |
| | | 61–120 | 25 | — | — | — | 25 |
| | M | 0–60 | 1 | — | — | — | 1 |
| | | 61–120 | 11 | — | — | — | 11 |
| 2 | F | 0–60 | 5 | — | — | — | 5 |
| | | 61–120 | 13 | — | — | — | 13 |
| | M | 0–60 | 6 | — | — | — | 6 |
| | | 61–120 | 13 | 1 | — | — | 12 |
| 5 | F | 0–60 | 8 | — | — | — | 8 |
| | | 61–120 | 69 | *5 | 2 | 14 | 48 |
| | M | 0–60 | 1 | — | — | — | 1 |
| | | 61–120 | 5 | *1 | — | — | 4 |
| 10 | F | 0–60 | 0 | — | — | — | — |
| | | 61–120 | 5 | **1 | 1 | 2 | 1 |
| 20 | F | 0–60 | 10 | 3 | 2 | 1 | 4 |
| | | 61–120 | 13 | 4 | *6 | 1 | 2 |
| 50 | F | 0–60 | 12 | 4 | 6 | 1 | 1 |

* Including 1 sarcoma.
** One carcinoma with glandular features.

dying in the first 60 weeks and the second 60 weeks of the various experiments. Cyst agglomerates were often recognisable to the naked eye and measured up to 2 cm. in diameter. They were multilocular and might replace extensive portions of the liver parenchyma. The contents of the larger ones were gelatinous. The cysts were lined with flat or cuboidal epithelial cells and might contain a pale eosinophilic PAS negative material. Septa between the cysts were thin but some papillary formation might be seen. Remnants of liver cords were often seen at the periphery and there was no capsule (Fig. 3 and 4). No nuclear irregularities were observed. Small isolated subcapsular cysts (Fig. 5) may be found in both experimental and in untreated rats and are considered to be distinct from the agglomerates. While the livers of rats on 20 and 50 p.p.m. DMN were grossly distorted with multiple cysts and tumours, these usually occurred singly in rats on lower doses. When cysts and tumours occurred together in the same liver the lesions were quite distinct. Of the 88 rats that received 5 or 10 p.p.m. DMN 19 had cyst agglomerates but only 3 had liver tumours as well; on the other hand of the 9 rats with tumours only 3 also had cystic agglomerates. Thus these lesions, while associated with an exposure to DMN, seem to develop independently.

Hyperplastic nodules (Fig. 6) were seen in 9 rats and in 7 there were also frank liver tumours making the differential diagnosis difficult but not of particular importance. Frank hepatomas were seen in 36 rats and the incidence was clearly dose-dependent in spite of the fact that animals on the lower doses lived longer. Of the tumours 33 were carcinomas of the trabecular or anaplastic type. Haemorrhage and areas of necrosis were common and metastases were found in the lungs of 11 rats. In 3 rats the tumours were spindle cell sarcomas with haemorrhage and necrosis but no metastases were found.

*Kidney*

No renal tumours were seen. The characteristic senile changes (Magee, 1959) were noted in rats which survived for more than a year. In 42 rats enlarged tubules similar to those described by others (Allen, Fisher and Adams, 1964; Foley *et al.*, 1964, and Terracini, Palestro, Gigliardi and Montesano, 1967) were observed. These were lined by large epithelial cells with pale uniformly staining cytoplasm and round nuclei. Small papillae were sometimes seen (Fig. 7). These lesions appeared irrespective of the sex or treatment of the rats and were common in the older rats, although not always associated with the senile changes.

*Lungs*

In addition to the inflammatory changes and associated bronchiectasis and liver tumour metastases, a benign primary tumour was found in 4 rats on 2 or 5 p.p.m. DMN. In one a papilloma obstructed a bronchus of a female which also had a breast tumour. In 3 rats there were small adenomas replacing lung parenchyma with the formation of irregular cavities lined with cuboidal cells with large nuclei. Although they had no capsule there were no histological signs of malignancy. The tumours were quite distinct from the hyperplasia seen associated with chronic inflammation.

*Other tumours*

The incidence of other tumours is given in Table III. There is no obvious relationship between the site and frequency and the dose of DMN which the animals had received.

---

EXPLANATION OF PLATES

FIG. 1.—Rat (F). DMN 20 p.p.m. for 50 weeks. Liver: enlarged and confluent portal spaces with gland formation. H. & E. × 125.

FIG. 2.—Rat (F) control killed 115th week. Small cluster of glandular cavities lined by cuboidal cells in a portal space. H. & E. × 125.

FIG. 3.—Rat (F). DMN 5 p.p.m. for 110 weeks. Liver: circumscribed cystic lesion. Cysts are empty, irregularly shaped and intermingled with liver parenchyma. H. & E. × 50.

FIG. 4.—Rat (F). DMN 5 p.p.m. for 111 weeks. Liver: cyst agglomerate; cysts lined by flat epithelium are separated by thin fibrous septa. No atypical cells. H. & E. × 140.

FIG. 5.—Rat (F). DMN 5 p.p.m. for 104 weeks. Liver: isolated cyst lined by flat epithelial cells. This was the only change found in the liver. H. & E. × 140.

FIG. 6.—Rat (F). DMN 20 p.p.m. for 61 weeks. Liver: well defined nodule composed of sheets of liver cells with no topographical relations to vascular structures or portal spaces. No atypical cellular features or mitoses. This animal had no liver tumours. H. & E. × 55.

FIG. 7.—Rat (F). DMN 2 p.p.m. for 95 weeks. Kidney: hyperplastic tubule lined by tall cells with pale cytoplasm. Some nuclei are piled up. P.A.S. × 140.

1

2

3

Terracini, Magee and Barnes.

4

5

Terracini, Magee and Barnes.

6

7

TABLE III.—*Kidney Lesions and Incidence of Other Tumours in Rats Receiving DMN*

| DMN p.p.m. | Sex | No. | *Renal tubule hyperplasia | Lung tumours | Mammary tumours | Lymphoma | Other tumours |
|---|---|---|---|---|---|---|---|
| 0 | M | 12 | 2/11 | 0 | 0 | 1 | 1 subcutaneous tumour (no histology). |
| 0 | F | 29 | 6/26 | 0 | 3 | 0 | 1 uterine sarcoma. 1 pyloric adenocarcinoma. |
| 2 | M | 19 | 4/18 | 1 | 0 | 1 | 1 carcinoma of mouth. |
| 2 | F | 18 | 7/12 | 2 | 3 | 0 | 1 adenocarcinoma of ovary. |
| 5 | M | 6 | 3/5 | 0 | 0 | 0 | — |
| 5 | F | 77 | 17/72 | 1 | 5 | 3 | 1 carcinoma of mouth. 1 intestinal sarcoma. 2 uterine adenocarcinomas. 1 pyloric adenocarcinoma. 1 peritoneal sarcoma. 1 ovarian tumour. |
| 10 | F | 5 | 0/5 | 0 | 0 | 0 | 1 carcinoma of mouth. |
| 20 | F | 23 | 2/20 | 0 | 0 | 0 | 1 peritoneal sarcoma. |
| 50 | F | 12 | 0/9 | 0 | 0 | 0 | — |

* Number of animals showing the lesion from among those from which kidney sections were available.

### DISCUSSION

The exposure of rats to low doses of DMN throughout their life span was undertaken in order to try to establish a sequence of pathological changes that might run parallel with the change in tumour incidence. Since the whole liver of a rat exposed to high doses of DMN undergoes a plethora of histological and cytological changes in association with the development of tumours, it was hoped that there might be some widespread non-cancerous changes which would provide evidence of possible impending malignant transformation. The comprehensive study of the liver changes in the rats exposed to low doses of DMN revealed no such indication. While the incidence of tumours fell rapidly when the dose was decreased from 50 to 5 p.p.m., some malignant tumours were seen at the latter dose and even at 2 p.p.m. The only other liver changes consistently found were cyst agglomerates; these were somewhat more frequent than hepatomas in rats on 5 p.p.m. DMN and were not seen at all in those on 2 p.p.m. As indicated above, these cyst agglomerates appeared to arise quite independently from the hepatomas. It was not possible to detect cytological changes or minor deviation from normality in hepatic architecture that made it possible to distinguish rats on a diet containing 2 or 5 p.p.m. DMN that would produce a low incidence of tumours, and rats on a normal diet of which none in our laboratory has developed hepatomas.

It is very important to try to establish some idea of the dose-response relationships to the exposure of animals to carcinogens. However, in most laboratories it is impractical to expose very large numbers of animals to a carcinogen at doses that may be expected to produce only a very low incidence (say 1 : 1000) of tumours. Such an incidence would of course represent an appalling hazard to human populations. Druckrey (1952) has already expressed the view that all doses of carcinogens are purely additive but attempts to establish the truth of this at low dose levels of a carcinogen are frustrated by the limited life span of the experimental animals. The use of specific pathogen-free rats and mice with a longer life span will provide more opportunities for doing such studies. However, the simple concept of the additive effect of repeated doses is not applicable to every situation. The incidence of kidney tumours in rats given DMN illustrates such an exception.

Among rats surviving a single intraperitoneal $LD_{50}$ of DMN (6 mg.) 20% developed kidney tumours while a much higher incidence was seen in rats receiving a diet containing 200 p.p.m. DMN for 1 week (21 mg.) or 100 p.p.m. for 4 weeks (42 mg.) (Magee and Barnes, 1962). Furthermore, Jasmin and Riopelle (1964) found 100% incidence in rats given 1·5 mg. DMN daily for 6 days only (9 mg.). In the experiments described here no kidney tumours were found in rats receiving 5 p.p.m. DMN for up to 104 weeks (54 mg.). (The total dose of DMN given in parentheses is calculated on the basis of a daily intake of 15 g. of diet.) Thus the total dose of carcinogen ingested is not always the only factor in producing tumours. It is probable that the crucial factor is the amount of the carcinogen which reaches the site of action as has been accepted for other drug effects (Brodie, Cosmides and Rall, 1965). In the case of DMN which almost certainly has to be metabolised before it becomes carcinogenically active it has long been known that the metabolism of a second dose of DMN is slowed when it follows closely after the first dose (Magee, 1956). Under these circumstances it is easy to visualise an effective dose reaching the kidneys.

The main object of these long-term studies on the effect of low doses of carcinogens was to try to provide a basis from which to extrapolate a no-effect dose. The present work indicates that in the case of the liver and the active nitrosamines, morphological changes cannot provide a basis for recognising effects before the appearance of tumours. Any hope of establishing a no-effect dose of a carcinogen must rest upon finding out more about the biochemical changes that must precede the development of neoplasia with the hope of being able to establish a dose below which such biochemical disturbances do not take place.

### SUMMARY

1. The pathological changes in rats kept for a lifetime on diets containing 5 and 2 p.p.m. dimethylnitrosamine (DMN) are described.

2. While the incidence of liver tumours falls rapidly when the dietary concentration of DMN is reduced from 50 to 5 p.p.m. a single rat on 2 p.p.m. developed a liver tumour so that a no-effect level has not been established.

3. There were no pathological changes in the liver other than tumours which provided a more sensitive index of exposure to DMN.

4. No kidney tumours were seen in any rats and the dose response relationship for DMN and tumours of the kidney is discussed.

We wish to thank Mr. C. R. Kennedy for his constant attention to the animals and their diets during these experiments; Mr. R. Legg for the photographs and Mr. J. A. E. Jarvis for making the determination of DMN in the diets.

### REFERENCES

Allen, D. A., Fisher, H. and Adams, M.—(1964) *Archs Path.*, **77**, 268.
Brodie, B. B., Cosmides, G. J. and Rall, D. P.—(1965) *Science, N.Y.*, **148**, 1547.
Bruce, H. M. and Parkes, A. S.—(1957) *J. Anim. Techns Ass.*, **7**, 54.
Druckrey, H.—(1952) *Arzneimittel-Forsch.*, **1**, 383.
Foley, W. A., Jones, D. C., Osborn, G. K. and Kimeldorf, D. J.—(1964) *Lab. Invest.*, **13**, 439.
Heath, D. F. and Jarvis, J. A. E.—(1955) *Analyst, Lond.*, **80**, 613.
Jasmin, G. and Riopelle, J.-L.—(1964) *Revue can. Biol.*, **23**, 129.
Magee, P. N.—(1956) *Biochem. J.*, **64**, 676.—(1959) *Coll. Pap. Lab. Anim. Centre, Carshalton*, **8**, 59.
Magee, P. N. and Barnes, J. M.—(1956) *Br. J. Cancer*, **10**, 144.—(1962) *J. Path. Bact.*, **84**, 19.—(1967) *Adv. Cancer Res.*, **10**, 164.
Stewart, H. L. and Snell, K. C.—(1957) *Acta Un. int. Cancr.*, **13**, 770.
Terracini, B. and Magee, P. N.—(1964) *Nature, Lond.*, **202**, 502.
Terracini, B., Palestro, G., Gigliardi, M. R. and Montesano, R.—(1966) *Br. J. Cancer*, **20**, 871.